# Molecular Mechanism Exploration of Autologous Blood Transfusion with RBC Surface Membrane Protein pMHC/aCD28 Combined with CD8+T Cells to Promote the Proliferation of CD8+T Cells to Inhibit the Malignant Transformation of Liver Cancer

**DOI:** 10.1155/2022/6102672

**Published:** 2022-09-28

**Authors:** Haiyong Tao, Tong Liu, Na Yao, Jinhuo Wang, Jiaming Xu, Xiaofang Zhou, Xiaofei Chen, Jianrong Guo

**Affiliations:** ^1^Department of Anesthesiology, Shanghai Gongli Hospital, Naval Military Medical University, Shanghai 200135, China; ^2^Graduate School of Wannan Medical College, Wuhu 241002, Anhui, China; ^3^Ningxia Medical University, Gongli Hospital of Shanghai Pudong New Area Training Base, Shanghai 200135, China; ^4^Department of Anesthesiology, Lihuili Hospital, Medical School of Ningbo University, Ningbo 315040, Zhejiang, China

## Abstract

Autologous blood transfusion is an important blood protection measure. Red blood cells have a certain degree of immunogenicity and their surface membrane proteins CD28 and MHC can participate in the immune response and interact with CD8+ T cells. We build a cell model with a transwell system. The binding characteristics of RBCs and CD8+ T cells were observed with a fluorescent confocal microscope. The content of the inflammatory factor TNF-*α* and IFN-*γ* produced was analyzed by ELISA. The proliferation characteristics of CD8+ T cells were analyzed by CFSE staining, and the content of CD3+CD8+ T cells was analyzed by flow cytometry. Cell migration and invasion experiments were used to analyze the malignant metastasis ability of liver cancer cells. The expression of vimentin, E-cadherin, and *β*-catenin was analyzed by Western blot. We establish a liver cancer model in rats and group them for autologous blood transfusion. The content of CD3+CD8+T cells in the blood of each group of rats was analyzed by flow cytometry. Western blot was used to analyze the expression of vimentin, E-cadherin, and *β*-catenin in the liver tissues of rats in each group. The red blood cells in the autologous reinfusion blood and CD8+ T cells have an obvious combination. The degree of combination of the two is related to the expression of CD28 and MHC. If CD28 and MHC are expressed at the same time, the combination of the two cells will be high, the proliferation of CD8+ T cells will increase, and the expression of inflammatory factors will also increase, while the expression of the three proteins that are positively correlated with the activity of cancer cells will decrease. If only one of CD28 and MHC is normally expressed, the result is contrary to the situation where both membrane proteins are normally expressed. Our project has proved that autologous infusion of red blood cell surface membrane proteins CD28 and MHC combined with CD8+ T cells can promote the proliferation of CD8+ T cells to inhibit the malignant transformation of liver cancer.

## 1. Introduction

Autologous blood transfusion is an important blood protection measure. At present, there are three types of autologous blood transfusion methods: preoperative autologous blood collection and storage technology, acute normovolemic blood dilution, and perioperative blood recovery technology [1]. The most commonly used is the third type of recovery type autologous blood transfusion in surgery. In the surgery process, through a negative pressure suction device, the bleeding during the operation is recovered to the blood storage tank and an appropriate amount of anticoagulant is mixed during the suction process. After multilayer filtration, the red blood cells are separated by a high-speed centrifugal pump, and the waste liquid, broken cells, and harmful components are diverted to the waste liquid bag. Finally, the red blood cells can be washed and concentrated with normal saline to obtain concentrated red blood cells with a hematocrit of 50–65%, which are collected in a blood storage bag and returned to the patient [2].

Red blood cell immunity is a defense mechanism of the body, and red blood cells have many immune-related substances. It is closely related to other immunologically active cells such as T lymphocytes, B lymphocytes, natural killer cells, and phagocytes [3]. There is a protein called complement receptor (CR1) on the surface of red blood cells, which can adhere to immune complexes and is the main bearer of carrying, transporting, and clearing circulating immune complexes (CIC) in the body [4]. Red blood cell immunity mainly includes identifying and carrying antigen, eliminating circulating immune complexes, enhancing the immune response of T lymphocytes, and promoting the phagocytosis of immune complexes by macrophages. Red blood cells have peroxidase on the surface of their cell membranes, which plays the same killing effect on immune complexes as macrophages [5]. There are a large number of red blood cells; the number of red blood cells is more than 1,000 times the number of white blood cells. More than 80% of CR1 in the blood circulation exists on the surface of red blood cells, so the chance of red blood cells meeting circulating immune complexes is 500 to 1000 times greater than that of white blood cells [6]. The task of removing “junk” from the body is mainly undertaken by red blood cells, which is unmatched by white blood cells. Therefore, red blood cells have the ability to recognize, adhere, kill antigens, and eliminate CIC [7].

In recent years, studies have shown that red blood cells have a certain degree of immunogenicity and that the surface of red blood cells has the membrane protein pMHC/aCD28, which can bind to CD8+ T cells and promote CD8+ T cell proliferation [8, 9]. CD8+ T cell-mediated cytotoxicity can be induced not only through perforin-induced cell necrosis but also through perforin P granzyme to induce cell apoptosis [10].

## 2. Materials and Methods

### 2.1. In-Vitro Experiments

#### 2.1.1. Experiment Design

We construct a cell model system: cultivate red blood cells and CD8+T cells in the blood of patients with liver cancer in a sterile environment. We use the transwell culture system to carry out the experiment. The upper layer of the transwell culture dish is cultured with autologously recovered RBCs/CD8+T cells, and the lower layer is cultured with autologously recovered CD8+T cells. We divide the cells into three groups, one group with the upper layer left unchanged, one group with the upper layer added with CD28 antibody, and one group with the upper layer added with MHC antibody. We add CD3-PE and CD8-PerCP labelled antibodies to the lower layer of the co-culture system, and add Atto488 and ALexa647 fluorescent dyes to the upper layer. After 7 days of routine aseptic culture, the binding characteristics of RBCs and CD8+ T cells were observed with a fluorescent confocal microscope.

The red blood cells of the upper layer of each group were collected, and the expression characteristics of Atto488-MHC molecules and ALexa647-CD28 molecules on the surface of red blood cells were analyzed by immunofluorescence. The supernatant of each group was collected, and the content of the inflammatory factor TNF-*α* and IFN-*γ* produced was analyzed by ELISA. The CD8+ T cells in the lower layer of each group were collected, the proliferation characteristics of CD8+ T cells were analyzed by CFSE staining, and the content of CD3+CD8+ T cells was analyzed by flow cytometry.

The transwell system was used to culture liver cancer cells (lower layer) from the resected tissues of liver cancer patients aseptically, and co-cultured with CD8+ T cells (upper layer) after induction of proliferation for 10 days. Cell migration and invasion experiments were used to analyze the malignant metastasis ability of liver cancer cells. We collect the liver cancer cells in each group and analyze the expression of vimentin, E-cadherin, and *β*-catenin by Western blot.

#### 2.1.2. Immunofluorescence Analysis

For each group of cells, 1% bovine serum albumin (BSA) was applied for blocking at room temperature for 30 min to block nonspecific epitopes. We incubate the specific primary antibody according to the recommended instructions for the antibody and let it stand overnight in a humidified box at 4°C. Next day, we take out the wells and rewarm at room temperature for 30 min. We select the corresponding immunofluorescence secondary antibody and then incubate at 37°C for 30 min in the dark. We add an antifluorescence quencher for mounting. Finally, we use a fluorescence microscope to observe and take pictures.

#### 2.1.3. Enzyme-Linked Immunosorbent Assay (ELISA)

Five standard wells were set on the ELISA-coated plate. The standard samples were added to the wells according to the concentration requirements and serially diluted. The sample volume in each well was 50 *μ*l. Then, a blank control well and the sample well to be tested were set. The blank control well would not add sample and enzyme-labeled reagent; the other steps were the same as the sample well. Add 40 *μ*l of sample diluent to the sample well, and then add 10 *μ*l of the sample. The final dilution of the sample is 5 times. Then, we seal the plate with a sealing film and incubate at 37°C for 30 minutes. After incubation, we wash the plate with the washing solution and dry it. After drying, we add 50 *μ*l of enzyme-labeled reagent to each well except the blank wall. Repeat the incubation and washing steps. Then, we add 50 *μ*l of developer A and 50 *μ*l of developer B in each well sequentially, develop the color at 37°C for 15 minutes in the dark, and lastly add 50 *μ*l of stop solution to stop the reaction (the blue turns to yellow immediately). We set the blank control well as zero, and measure the absorbance (OD value) of each well in sequence at 450 nm wavelength.

#### 2.1.4. CFSE Fluorescence Staining

We dissolve CFSE (carboxyfluorescein succinimidyl amino ester) with dimethyl sulfoxide (DMSO) to 5 mmol/L and store at −20°C in the dark. When in use, it is diluted to 5 *μ*mol/L with serum-free Dulbecco's modified eagle medium (DMEM) medium as a working solution for later use. The lower cell culture medium of each group was used to separate the cells by centrifugation, and the cells were resuspended in the working solution to a concentration of 1 × 10^6^/ml. The resuspension was incubated at 37°C for 30 minutes, and 5 times the volume of cold medium was added to stop staining. Then, we incubate on ice for another 5 minutes, centrifuge to separate the cells, and rinse three times with fresh medium. Finally, the cells were resuspended in the culture medium, and the fluorescence intensity was detected by a flow cytometer under excitation light of 488 nm wavelength.

#### 2.1.5. Flow Cytometry Analysis

Cells from each group were added to a 2 ml centrifuge tube, centrifuged at 1500 rpm for 5 minutes, and the supernatant was discarded. We use 4% paraformaldehyde (PFA) to fix at 4°C for 30 min and then use 0.1% Triton X-100 to fix at room temperature for 10 min. We add 200 *μ*l of the primary antibody diluted with PBA, incubate at 4°C for 2 hours, then centrifuge to remove the supernatant and wash with PBS. Then, we add 200 *μ*l of fluorescein-labeled secondary antibody diluted with PBA and incubate for 30 min at 4°C in the dark. Finally, the cells were re-suspended in 500ul of PBS, placed in a flow tube, and detected by a flow cytometer.

#### 2.1.6. Cell Migration and Invasion Test

Twenty-four hours before the experiment, the cells of different groups were replaced with serum-free medium, and the culture was continued. Before inoculation, we soak the 24-well plate and transwell chamber with 1 × PBS for 5 min to moisten the chamber. We digest the cells, wash the cells with serum-free medium, resuspend the cells in serum-free medium, count the cells, and dilute to adjust the cell density to 5 × 10^5^/ml. then, we inoculate 0.2 ml cell suspension (5 × 10^4^ cells) into the transwell chamber, and then add 0.7 ml of RPMI-1640 medium containing 10% FBS to the lower 24-well plate, 3 replicate holes per group and place them in a 37°C incubator for 24 hours to terminate the culture. We add 1 ml of 4% formaldehyde solution to each well of the above cells and fix them at room temperature for 10 min. We aspirate the fixative solution and wash once with 1 × PBS. Then, we add 1 ml 0.5% crystal violet solution to each well and wash with 1 × PBS three times after dyeing for 30 min. We use a cotton swab to carefully wipe off the cells that have not migrated into the transwell, place them under a 200× microscope, and count the number of cells in each field of view.

After the cells were digested and counted, 8 × 10^5^ cells were divided into 35 mm^2^ culture dishes. We use a marker to draw a line on the bottom of the dish as a mark, aspirate the culture medium, and use a 10 *μ*l pipette tip to mark the cells in the dish perpendicularly to the marker. Then, we rinse with PBS to remove the marked cells, and add serum-free culture medium to continue culturing. We take pictures at 24 h, select the intersection of the line drawn by the marker and the cell scratch as the observation point, and then observe at a fixed point.

#### 2.1.7. Western Blot Analysis

After collecting cells from each group, we add 200 *μ*l of cell lysate to each six-well plate. After sonication, the cells were lysed on ice for 1 hour. The lysed cell sample was centrifuged at 12,500 rpm for 15 minutes at 4°C. Then, we transfer the supernatant in the centrifuge tube to a clean centrifuge tube. *β*-actin protein quantification kit was used to quantify protein concentration. The measured protein samples were stored at −80°C. In Western blot electrophoresis, the protein loading concentration was 50 *μ*g per well. After SDS-PAGE electrophoresis, the membrane was transferred and blocked. The primary antibodies against proteins vimentin, E-cadherin, and *β*-catenin (Thermo-Fisher, USA) were diluted to use concentration. The samples were incubated overnight on a shaker at 4°C. After washing with PBS, the samples were incubated with the secondary antibody (Thermo-Fisher, USA) for 30 minutes at room temperature in the dark. Finally, the developer was used for development and photography.

### 2.2. In-Vivo Experiments

#### 2.2.1. Experiment Design

We establish a rat model of liver cancer: buy 45 SD rats, and randomly divide them into 3 groups. One group did not establish a liver cancer model, and the other two groups were intraperitoneally injected with liver cancer cells HepG2 (1 × 10^6^ cells for 1 time), which were numbered as the normal control group, liver cancer model group 1, and liver cancer model group 2, respectively. The tumor formation of rats was observed and recorded every 3 days, and the model was established for 42 days to establish a rat model of liver cancer. Rats in the liver cancer model group underwent abdominal surgery to remove the macroscopic liver cancer tissue and the cancer tissue was preserved in liquid nitrogen for subsequent experiments. A domestic autologous-2000 blood recovery machine was used to recover blood from the operating field of each rat, and then the recovered red blood cells were labelled with Atto488 and ALexa647 fluorescent dyes.

Rat autologous blood transfusion: After liver cancer tissue resection was performed on the rats in the two model groups, each rat was injected with CD3-PE and CD8-PerCP-labeled antibodies to the tail vein. At the same time, rats in the NC group and model group 1 were intravenously injected with 10 ml of normal saline through the tail vein, and rats in model group 2 were infused with 10 ml of recovered autologous blood red blood cells. The three groups of rats were routinely fed for 8 weeks, and then the rats were sacrificed by cervical dislocation. The liver and lung tissues of each group of rats were collected, and the recurrence and metastasis of each group of rats were observed and recorded. The abdominal aorta blood of each group of rats was collected, and the content of CD3+CD8+T cells in the blood of each group of rats was analyzed by flow cytometry. A Western blot was used to analyze the expression of vimentin, E-cadherin, and *β*-catenin in the liver tissue of each group of rats.

#### 2.2.2. Flow Cytometry Analysis

The detailed steps of flow cytometry analysis are the same as those in the in-vitro experiments section, so they would not be repeated here.

#### 2.2.3. Western Blot Analysis

The detailed steps of Western blot analysis are the same as those in the in-vitro experiments section, so they will not be repeated here.

### 2.3. Statistical Analysis

The experimental results are expressed as a mean ± standard deviation. Statistical analysis was performed using Statistical Product and Service Solutions (SPSS) 22.0 software (IBM, Armonk, NY, USA). The figures were produced with Origin 2021 and Adobe Illustrator 2020 software.

## 3. Results

### 3.1. RBCs and CD8+T Cells Combined Together

**Figure 1:**
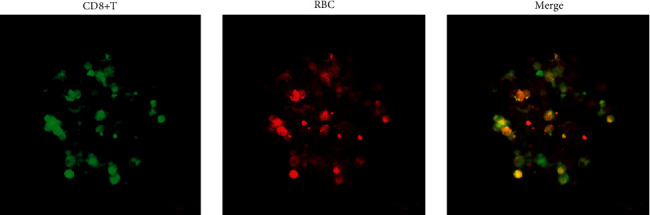
The results of RBCs and CD8+ T cells photographed by a confocal fluorescence microscope. There is a clear overlap between the two types of cells.

The results taken by a fluorescent confocal microscope are shown in Figure 1. It can be seen from the figure that by overlapping the two fluorescent images, the positions of CD8+ T cells overlap with RBC, indicating that almost all CD8+T cells are combined with RBC. This phenomenon confirmed the hypothesis that CD8+T cells bind to RBC through the CD28/MHC protein.

### 3.2. The Expression of CD28/MHC Is Inhibited by Their Corresponding Antibodies

**Figure 2:**
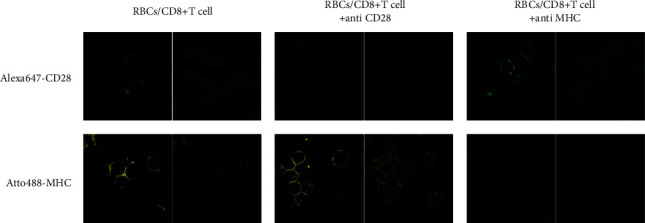
The results of immunofluorescence analysis of the expression characteristics of Atto488-MHC and ALexa647-CD28 molecules on the surface of red blood cells. The amount of molecule expression is directly proportional to the fluorescence intensity.

The results of immunofluorescence analysis of the expression characteristics of Atto488-MHC and ALexa647-CD28 molecules on the surface of red blood cells are shown in Figure 2. It can be seen from the figure that the expression of CD28 in the group with CD28 antibody decreased significantly, while the expression of CD28 in the other two groups were more similar. The expression characteristics of MHC in the group with MHC antibodies and the other two groups are consistent with CD28. This shows that CD28 antibody and MHC antibody can effectively inhibit the expression of CD28 and MHC, which provided an effective means of controlling variables for the experiment.

### 3.3. Stronger Inflammatory Response Occurred with the CD28/MHC Simultaneous Expression

**Figure 3:**
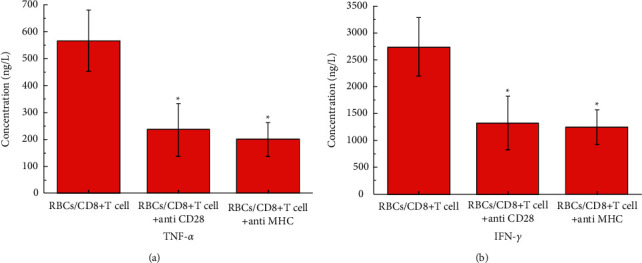
The results of ELISA analysis of the content of inflammatory factors TNF-*α* and IFN-*γ*. The symbol ^*∗*^means *P* < 0.05 (compared to RBCs/CD8+T cell group).

The results of ELISA of the content of inflammatory factors TNF-*α* and IFN-*γ* are shown in Figure 3. It can be seen from the figure that when CD28 and MHC are expressed at the same time, the contents of inflammatory factors TNF-*α* and IFN-*γ* are significantly higher than when the other two groups of CD28 or MHC are expressed alone. Inflammatory factors are proteins expressed by immune cells that trigger an inflammatory response. High expression of inflammatory factors means a large number of immune cells' proliferation. This shows that CD8+T cells can proliferate more effectively when CD28 and MHC are present at the same time.

### 3.4. CD8+T Proliferated with the CD28/MHC Simultaneous Expression

**Figure 4:**
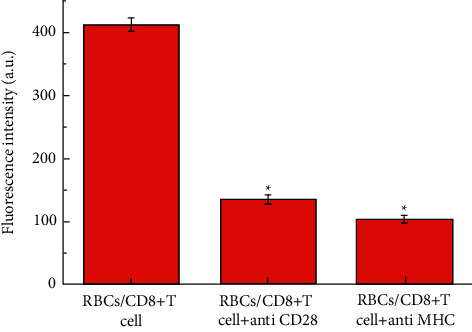
The results of CFSE staining to analyze the proliferation characteristics of CD8+ T cells. The proliferation of CD8+ T cells is positively correlated with fluorescence intensity. The symbol ^*∗*^means *P* < 0.05 (compared to RBCs/CD8+T cell group).

The results of CFSE staining to analyze the proliferation characteristics of CD8+ T cells are shown in Figure 4. The fluorescence intensity in the figure represents the proliferation of CD8+ T cells. It can be seen from the figure that when CD28 and MHC are expressed at the same time, the proliferation of CD8+ T cells is significantly greater than when the other two groups of CD28 or MHC are expressed alone. This shows that CD8+ T cells can proliferate more effectively when CD28 and MHC are present at the same time. The results of this experiment are consistent with the results of the ELISA.

### 3.5. Proportion of CD3+CD8+ T Cells Increased with the Proliferation of CD8+ T Cells

**Figure 5:**
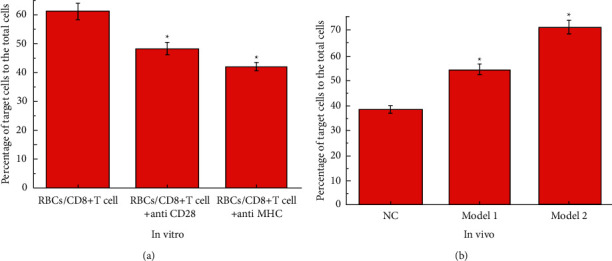
The results of flow cytometry analysis of CD3+CD8+T cell content. (a) The results of the in vitro experiment. The symbol ^*∗*^means *P* < 0.05 (compared to RBCs/CD8+T cell group). (b) The results of the in vivo experiment. The data of the NC group are consistent with the normal value. The symbol ^*∗*^means *P* < 0.05 (compared to the NC group).

The results of flow cytometry analysis of CD3+CD8+T cell content in in vitro and in vivo experiments are shown in Figure 5.  Figure 5(a) shows the results of in vitro experiments. CD3+CD8+ T cells are killer T cells, which have a direct killing effect on cancer cells. It can be seen from the figure that when CD28 and MHC are expressed at the same time, the proportion of CD3+CD8+ T cells is significantly higher than that of the other two groups. This shows that when the proliferation of CD8+ T cells increases, the ratio of CD3+CD8+T cells also increases. That is, it can kill cancer cells more effectively.

Figure 5(b) shows the results of the in vivo experiment. It can be seen from the figure that compared with model group 1, the proportion of CD3+CD8+ T cells in model group 2 is higher, which means that the proliferation of CD8+ T cells is greater. This shows that autologous red blood cell reinfusion increases the proliferation of CD8+ T cells, enabling the immune system to deal with liver cancer more effectively.

### 3.6. Lowest Cancer Cell Activity Existed with the CD28/MHC Simultaneous Expression

**Figure 6:**
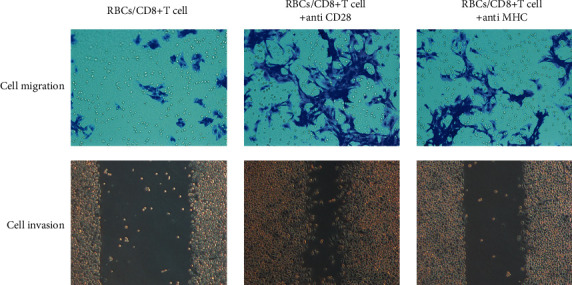
The results of cell migration and invasion experiments.

The results of cell migration and invasion experiments are shown in Figure 6. Both cell migration and invasion experiments are commonly used methods to determine the ability of cancer cells to proliferate and metastasize. The greater the number of cell migration and the narrower the scratch, the greater the activity of cancer cells and the stronger the ability of proliferation and malignant metastasis. It can be seen from the figure that the migration and invasion of cancer cells in the group where CD28 and MHC are expressed at the same time are the weakest, indicating that the proliferation of CD8+ T cells is the largest, which is consistent with the results of the CFSE staining experiment.

### 3.7. Analyzed Protein Expression Positively Correlated with the Activity of Cancer Cells

**Figure 7:**
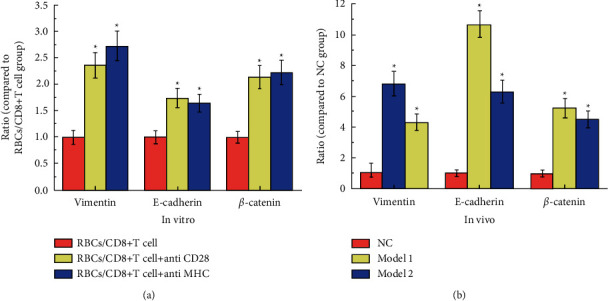
The results of western blot analysis of protein vimentin, E-cadherin, and *β*-catenin expressions. (a) The results of the in vitro experiment. The symbol ^*∗*^means *P* < 0.05 (compared to RBCs/CD8+T cell group). (b) The results of the in vivo experiment. The data of the NC group are consistent with the normal value. The symbol ^*∗*^means *P* < 0.05 (compared to the NC group).

The results of Western blot analysis of proteins vimentin, E-cadherin, and *β*-catenin expression in in vitro and in vivo experiments are shown in Figure 7. Vimentin is one of the proteins in the intermediate filaments, which form the cytoskeleton together with microtubules and actin microfilaments. E-cadherin participates in cell-cell adhesion and plays an important role in maintaining cell polarity and integrity. *β*-catenin acts as a transcription factor in the nucleus and activates genes that promote cell division. The expression of these three proteins is positively correlated with the activity of cancer cells.

Figure 7(a) shows the results of in vitro experiments. It can be seen from the figure that when CD28 and MHC are expressed at the same time, the expression of the three proteins is significantly lower than the other two groups, which means that cancer has been suppressed to a greater extent.  Figure 7(b) shows the results of the in vivo experiment. It can be seen from the figure that the expression levels of the three proteins in model group 2 of autologous red blood cell reinfusion were lower than those in model group 1, which once again confirmed the effect of autologous reinfusion of red blood cells to activate the proliferation of immune cells CD8+T.

## 4. Discussion

This subject established scientific and rigorous in vitro and in vivo models and adopted strict variable control methods to study the relationship between autologous reinfusion of red blood cells and CD8+ T cell proliferation [11]. Through basic and effective cell experiments and biochemical characterization methods, it has been proved that the membrane proteins CD28 and MHC on the surface of autologous reinfused red blood cells can bind to CD8+T cells, thereby promoting the proliferation of CD8+T cells and inhibiting the malignant transformation of liver cancer.

CD8+ T cells are an important member of the cytotoxic T lymphocyte (CTL) family. It is a subdivision of white blood cells and is a specific T cell that secretes various cytokines to participate in immunity. It has a killing effect on certain viruses, tumor cells, and other antigenic substances, and forms an important line of defence for the body's antivirus and antitumor immunity with natural killer cells [12–15]. TNF-*α* and IFN-*γ* are two inflammatory factors that play a major role after CD8+ T cells are activated. As a major tumor necrosis factor, TNF-*α* plays a key role in killing cancer [16]. IFN-*γ* has a wide range of immunomodulatory effects and is one of the main cofactors in suppressing cancer [17]. In this experiment, the two inflammatory factors and the content of CD8+ T cells were compared successively to determine the tremendous influence of CD28 and MHC on the autologous infusion of red blood cells on the proliferation of CD8+ T cells.

CD3+CD8+T cells, as a subtype of the CD8+T cell family, have a strong killing effect on cancer cells in the body [18]. In this experiment, as the proliferation of CD8+ T cells increases, the proportion of CD3+CD8+ T cells in the total cells also increases, which means that the immune system can inhibit and kill liver cancer more effectively.

Vimentin is one of the proteins in the intermediate filaments, which form the cytoskeleton together with microtubules and actin microfilaments. Vimentin is mainly attached to the side or end of the nucleus, endoplasmic reticulum, and mitochondria, so it is considered to play an important role in supporting and anchoring the organelles in the protoplasm. It can also maintain the shape of the cell and the integrity of the cytoplasm and stabilize the interactions within the cytoskeleton [19]. E-cadherin is a calcium-dependent transmembrane protein, which is widely distributed inside and outside various epithelial cells of humans and animals. It is mainly involved in cell-cell adhesion and plays an important role in maintaining cell polarity and integrity [20]. *β*-catenin is an important regulatory protein of the WNT signaling pathway. It binds to the intracellular part of E-cadherin in resting cells to stabilize cell adhesion. In cells activated by WNT signaling, *β*-catenin enters the nucleus from the cytoplasm to become a transcriptional activator [21]. These three proteins all play an important role in the process of malignant transformation of liver cancer, and their expression levels are obviously positively correlated with cancer activity [22]. In this experiment, the relative expression levels of the three proteins were used as the main indicators for judging cancer activity to determine the effectiveness of CD8+ T cell proliferation.

## 5. Conclusion

In summary, our project has successfully proved through a series of experiments that autologous infusion of red blood cell surface membrane proteins CD28 and MHC combined with CD8+ T cells can promote the proliferation of CD8+ T cells to inhibit the malignant transformation of liver cancer. Although there are still many shortcomings in the research process of this subject, more in-depth research is needed to make more progress. This subject still provides a new strategy for the diagnosis and treatment of liver cancer.

## Data Availability

The datasets used and analyzed during the current study are available from the corresponding author upon reasonable request.
